# An annotation infrastructure for the analysis and interpretation of Affymetrix exon array data

**DOI:** 10.1186/gb-2007-8-5-r79

**Published:** 2007-05-11

**Authors:** Michał J Okoniewski, Tim Yates, Siân Dibben, Crispin J Miller

**Affiliations:** 1 Bioinformatics Group, Cancer Research UK, Paterson Institute for Cancer Research, The University of Manchester, Christie Hospital Site, Wilmslow Road, Manchester M20 4BX, UK; 2 Molecular Biology Core Facility, Cancer Research UK, Paterson Institute for Cancer Research, The University of Manchester, Christie Hospital Site, Wilmslow Road, Manchester M20 4BX, UK

## Abstract

Affymetrix exon arrays contain probesets intended to target every known and predicted exon in the entire genome, posing significant challenges for high-throughput genome-wide data analysis. X:MAP , an annotation database, and exonmap , a BioConductor/R package, are designed to support fine-grained analysis of exon array data. The system supports the application of standard statistical techniques, prior to the use of genome scale annotation to provide gene-, transcript- and exon-level summaries and visualization tools.

## Abstract 2

An annotation database (X:MAP) and BioConductor/R package (exonmap) have been developed to support fine-grained analysis of exon array data.

## Rationale

Alternative splicing has been implicated in a wide range of human diseases, including neuropathological conditions such as Alzheimer's disease, cystic fibrosis, those involving growth and developmental defects, and many human cancers [1,2]. It is involved in diverse cellular processes, including apoptosis, invasion, angiogenesis and differentiation [1], and can impact on both the efficacy and the toxicology of drugs [3].

Given that 40% to 60% of all human genes, corresponding to approximately 70% of all multi-exon genes, are predicted to be alternatively spliced [4,5], the prospect of being able to investigate coordinated changes in gene expression at the level of individual isoforms is of significant interest. Recently, a new generation of microarrays has been designed with substantially higher probe densities than were previously available, allowing probes to be targeted at individual exons and gene expression to be monitored at much finer granularities than before. The Affymetrix Exon 1.0 ST array, for example, has approximately 5.5 million features, corresponding to approximately 1.4 million probesets, targeting approximately 1.2 million individual exons. The aim was to design an array that interrogated every single known and predicted exon in the human genome.

**Figure 1:**
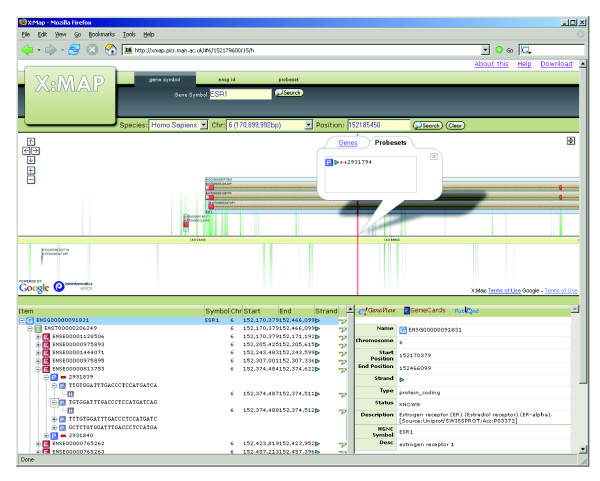
X:MAP displaying the 5' end of ESR1. The web interface can be searched by gene symbol, Ensembl ID, location or probeset ID. The central portion of the viewer is interactive and can be scrolled in real time by dragging with the mouse. Orange filled bars represent exons. UTRs are indicated by the white-filled portions of the exons. The tree in the bottom left panel displays, for the gene of interest, a hierarchy showing the relationship between genes, transcripts, exons, probesets, probes and their genome hit. Specificity of the probe-genome match is also displayed. The bottom right panel is context sensitive and is used to present detailed information for the selected item in the tree. These include hyperlinks to external annotation resources.

While such arrays offer great promise, they also pose major challenges for data analysis. Figure 1, for example, shows the transcript and exon structure for the 5' end of the oestrogen receptor (ESR1) gene accompanied by Affymetrix probeset target locations. The gene is represented by probesets targeting known exons, introns, putative exons, the untranslated regions (UTRs) and genomic sequences, both up- and downstream of the predicted gene. This level of coverage poses two problems: first, it must be possible to present and interpret such data for each individual gene of interest. Second, if analysis is to be pursued systematically (rather than simply by selecting genes based on prior knowledge), techniques for global, high-throughput, analyses of the entire expression dataset must also be available.

## Gene annotation is a graph

These challenges are compounded by the underlying complexity inherent in gene expression. In particular, the many-many relationships between genes, transcripts and exons define a graph structure that cannot be adequately represented as a table, and this is made more complex by the fact that a small but significant proportion of probes on the exon arrays (approximately 5%) are capable of hybridizing to multiple locations within the expressed genome [6]. This translates to >9% of probesets on the array.

Affymetrix microarrays use multiple probes targeting a particular region of interest, which are grouped in software to form a 'probeset'. In the initial stages of data processing, the signal from each probeset's constituent probes is combined in order to provide a summary value for that probeset. A variety of algorithms exist to do this, but all offer some kind of weighted or trimmed average, often moderated by an estimate of background signal. While Affymetrix offer their own probeset definitions, a number of alternative annotation strategies have been developed (see [7], for example). Although there is some variation in implementation, the overall approach is common: to map probe locations to a database of annotation, and to use this information to define new probesets based on gene, transcript or exon structure. Once these new probesets have been defined, they can be used to provide summary expression data. For example, a gene-centric probeset definition file might be generated in which a single monolithic probeset is created for each gene. Although this loses information at the level of individual exons, it offers the opportunity of providing gene-level data similar to those presented by 'traditional' expression arrays.

One issue that arises with such an approach is that in the design of their arrays, Affymetrix have included probesets that target exons predicted with different levels of confidence. Monolithic gene/transcript probesets must, therefore, either include low-confidence probes, with the potential to incorporate a significant number of false positives, or reject them, potentially missing putative coding regions and novel or previously uncharacterized genes or exons. In addition, not all transcribed RNA forms part of the coding sequence of a protein, and genes can overlap or contain one another. There are many non-coding genes, including rRNAs, tRNAs, small nucleolar RNAs (snoRNAs) and microRNAs, substantial antisense transcription, and even the possibility of low-level 'leaky transcription' [8-10]. At the time of writing a total of 31,718 genes are represented in the Ensembl human database, of which 11,241 (35%) have more than one annotated transcript. This is smaller than the 40% to 60% estimated in [4,5], and suggests that there are likely to be many alternative splicing events without representation in the databases. Probeset annotation strategies based solely on current database content are likely to miss many of these. A significant amount of care must, therefore, be taken when grouping probes together on the basis of their proximity to a known gene.

## Post-summary analysis of exon array data

Rather than using annotated gene structure to define large probesets (that is, prior to expression summary), an alternative approach is to make use of the original small (4-probe) probesets defined by the manufacturer, and only apply additional annotation data after the expression summary step.

One advantage of this is that both high- and low-confidence probesets can be included in the initial analysis, since they can later be excluded if necessary on the basis of, for example, their actual expression levels in real data. Maintaining the standard probeset definitions also makes it easier to compare between different experiments, both at the annotation level (as discussed in the MIAME recommendations [11]), and at the level of raw data. This is particularly important since differences in probeset mappings have been cited as a contributory factor to the poor correspondence often reported between microarray experiments [6,12,13]. Recently, it has also been shown that high levels of reproducibility can be found between Exon 1.0 ST arrays and the previous generation of HGU133plus2 chips, when attention is paid to the location of the individual probesets forming the mappings between arrays [6].

Thus, the approach described here utilizes a strategy in which probe sequences were mapped by *in silico *search to the entire genome, and associated with an annotation database. Existing probeset definitions are used, and detailed annotation only employed following expression summary. In the next two sections, the software is described, followed by an exemplar study.

## X:MAP, a database of fine-grained annotation for Affymetrix exon arrays

X:MAP is an annotation database built by mapping Affymetrix probeset sequences to the entire human genome, using a hashing approach similar to that described in [14,15]. These data are stored in a relational database implemented using mySQL, and associated with a local copy of the Ensembl database [16]. Probeset mapping code is implemented in Java, while database population is coordinated by a series of scripts that manage data download, probeset definition file parsing and database updates. For Ensembl build Homo Sapiens core 40 36 b, the software identifies 23,555,980 genome hits, from 5,467,261 probes that together form 1,432,150 probesets. On a dual processor 64 bit Linux workstation the entire process, including database updates, takes about 3 hours and generates about 1.2 GB of mapping data. In order to support local, or site-wide, installations, SQL data are available for download. In total, a local installation to support human arrays is approximately 16 Gb, including both Ensembl, and the additional mapping tables and indexes. Although large, this is comparable in size to the amount of raw data generated by a single exon array project, where each sample generates approximately 1.1 Gb of data (including DAT files), and is still of a reasonable size that can be accommodated by a modern workstation.

## The X:MAP genome browser

A challenge with genome browsing software is to provide an interface that responds fast enough to allow efficient interaction and browsing (Figure 1). The X:MAP interface makes use of the Google maps API [17] to provide a fully interactive, real time scrollable representation. The web interface is provided via tomcat and also allows the database to be searched according to gene name/symbol, transcript- or exon-id, and by genomic location. A hierarchical view of the gene-transcript-exon-probeset relationship is provided, along with hyperlinks to a variety of external annotation resources. A publicly accessible installation is available at [18].

## exonmap, a BioConductor/R package for Exon array analysis

An associated client-side BioConductor/R [19] package can connect to the database and execute a series of queries that allow associations between probesets, exons, genes and transcripts to be identified. Since many of these queries involve combining data extracted from multiple tables within the database, they are implemented as stored procedures on the database server. This results in much smaller data-transfer overheads between server and client, lower client-side memory requirements and makes use of the database infrastructure, which is specifically optimized for these tasks. It was the requirement for tightly coupled low-level access to both fine-grained (that is, exon level) genome annotation and probeset location data that led to the development of a single integrated database rather than building a database containing only probe and probeset hits, and making use of another external annotation resource such as EnsMart [20], BioMart [21] or ElDorado [22].

To the user, exonmap presents a set of functions of the form *X.to.Y() *(for example, *probeset.to.gene()*) that allow cross mappings to be made freely between probesets, exons, transcripts and genes. In addition, filtering functions are provided to include or exclude probesets based on the features they hit, and a variety of visualization functions to map and plot probeset expression levels as a function of gene-structure. These operations are described in more detail in the example below. Together they allow different features of the data to be explored, such as those characterized in Table 1, or to be mapped onto individual genes, and related to probeset expression, as in the gene plots.

**Table 1:** Number of features identified

List		Total no.
1	DE probesets*	11,109
2	Unique genes	1,507
3	Exons with ≥1 DE probeset	7,589
4	Genes from list 2 with CV of their exons >0.5	835
5	Novel expressed regions	2,125
6	Novel expressed regions outside known genes	1,170
7	Novel expressed regions within known genes	955
8	Genes with putative exons	382

## Overview of operation

**Figure 2:**
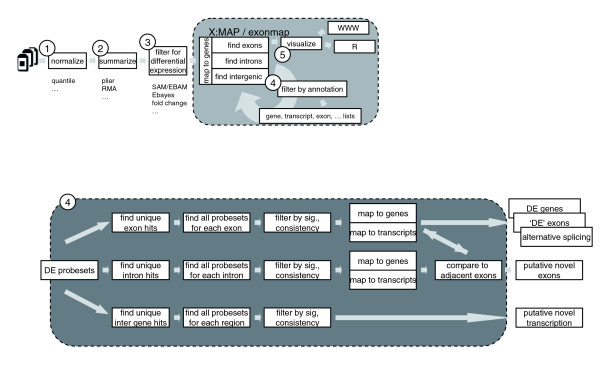
Use of the exonmap infrastructure to analyse exon microrrays. A processing pipelines using exonmap and X:MAP software. The first steps (1-3) are similar to standard microarray analysis. Step 4 is specific for exonmap, and is a combination of filtering and mapping between genes, transcripts, exon and probesets using genomic annotations from X:MAP. Step 5 comprises gene visualizations (see Figures 3 and 4).

An analysis pipeline such as that shown in Figure 2 may be used. Steps 1, 2 and 3 (expression summary, normalization, and identification of differentially expressed probesets) are identical to those that might be employed for standard expression arrays. Since the focus of this article is on annotation, we do not focus on the different strategies available for generating initial expression summaries, or for using these to identify a list of differentially expressed probesets (that is, steps 1, 2 and 3). It is our experience that current techniques work well, although particular care must be taken with multiple testing correction, given the large number of probesets represented on an exon array and their non-independence.

**Figure 3:**
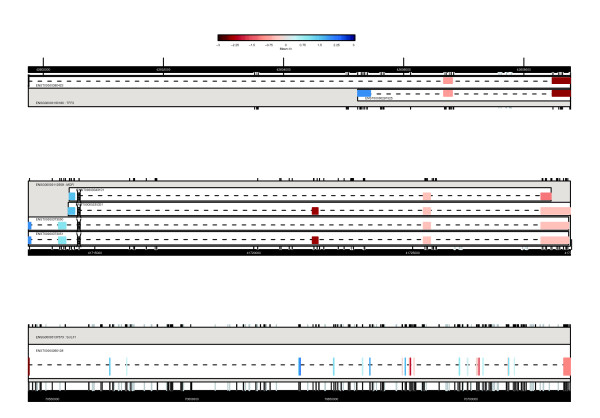
Examples of expression plots. Changes in differential expression between MCF7 and and MCF10A cell lines. Each plot represents a gene, and provides gene transcript and exon structure similar to that presented in the X:MAP web interface. Exon data are colored by mean fold-change between MCF7 and MCF10A, and scaled relative to the mean fold change for the entire gene. Only exon-targeting probesets uniquely hitting each gene are used for the calculations. Empty boxes with a cross through them represent exons that do not have a well behaved probeset targeting them with all probes.

**Figure 4:**
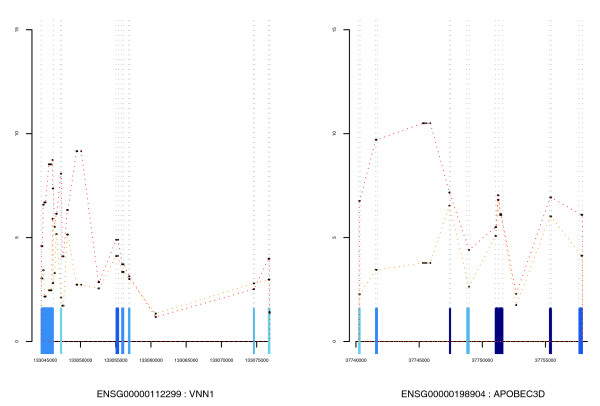
Examples of expression graphs. Line plots of individual probeset expression against genome location. All annotated exons are plotted along the x axis and colored as in Figure 3. These examples are taken from *list 7 *and represent genes containing intron-targeting probesets with significant behavior. The line plot includes these intronic probesets and allows their expression to be placed in the appropriate genomic context.

Once an appropriate probeset list has been generated, database queries are used to map probesets to genome structure. R functions exist to provide bi-directional mappings between probesets, genes, transcripts and exons. These can be used to identify genes where at least one probeset is differentially expressed (step 4), and then to provide full data for each of these genes (step 5). The software allows journal quality cartoons to be generated depicting the genomic structure of the region of interest and colored according to, for example, fold-change or expression level (Figures 3 and 4); alternatively, functions are provided to invoke an instance of a web browser targeted at the X:MAP query page and centered on the gene of interest (Figure 1).

## An example

Two cell lines were compared in triplicate, the human breast cancer cell line MCF7, and the non-tumorigenic breast epithelial cell line, MCF10A, which, unlike MCF7, lacks tumorigenicity in nude mice, three-dimensional growth in collagen, and spontaneous- and anchorage-independent growth. Full protocols are available in Additional data file 1, and data may be downloaded from [23].

In the following analysis, differentially expressed probesets were identified using SAM [24], as implemented in the siggenes package in BioConductor. We use SAM here because it is a popular and well understood method that provides a starting point from which to explore the database. Other approaches can clearly be substituted, but a discussion of their relative merits for exon array data is outside the scope of this article. It should be noted in passing, however, that the large number of non-independent probesets that are found within exon array data (since many genes are targeted by multiple probesets) pose significant challenges for algorithms that estimate false discovery rates (FDRs), or that correct for multiple testing. The infrastructure described here provides a possible source of annotation data with which to better inform these algorithms.

Raw expression data were processed in R using the affy BioConductor libraries. Expression summarization was performed using RMA [25,26] with chip definitions supplied via a custom CDF file that included all probesets originally defined by Affymetrix, but with background and control probesets removed. These can also be downloaded from the X:MAP website.

## Identifying differentially expressed known genes

The X:MAP database was then used via the exonmap R package to map these probesets to exons and to the genes that contain them. This mapping is achieved using an R function that calls a stored procedure on the database server. Implementation details are hidden from the user; the API simply provides a set of functions of the form *X.to.Y(<ID:String[]>) *that handle the intricacies of database IO and connections internally. For example, the function *probeset.to.gene(probestid) *takes a list of probeset IDs and returns a corresponding gene list, and was used to generate a list representing genes for which one or more probesets is differentially expressed. With a FDR of 8.1%, 11,109 probesets (these data are subsequently referred to as *list 1*) were identified in this way. Other options include mapping to introns (that is, include matches to regions not identified as containing an Ensembl exon, but within the extent of a known gene), transcripts, and to intergenic regions (discussed below). By choosing to include/exclude intron and intergenic matches, an explicit decision can be made as to whether to focus on well characterized regions. It is also important to apply a filtering to remove probesets containing probes capable of hybridizing in multiple locations; functions are included to filter probesets based on their specificity. When only matches between 'well behaved' probesets and known genes or exons are considered, the 11,109 Differentially Expressed (DE) probesets identified above map to 1,507 unique genes (henceforth, *list 2*) and 7,589 unique exons (*list 3*). All data are summarized in Table 1. By treating *list 2 *as a list of putative genes in which at least one exon is changing in 'expression' between replicate groups, it is possible to pursue them in more detail.

A number of strategies exist for doing this [27]. Here we use a variation of the 'splicing index' described in [28] to prioritize genes according to effects size. Other approaches, such as splicing ANOVA (MIDAS) [27] and ANOSVA [29], can be used similarly to select or prioritize based on statistical significance.

**Figure 5:**
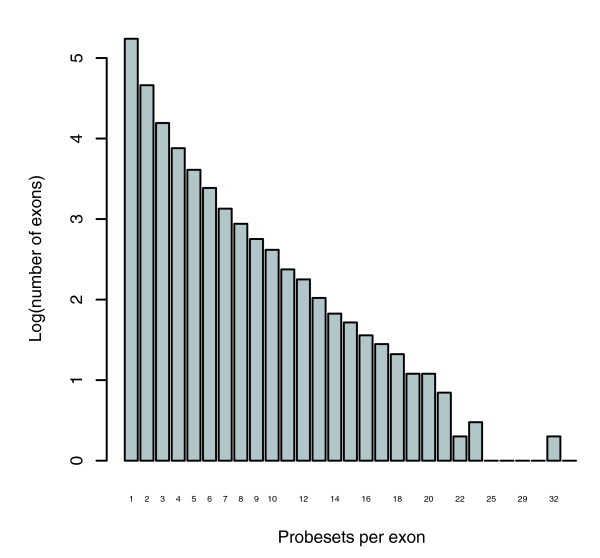
Histogram of number of probesets per exon. Data are on a log_10 _scale. Most exons are targeted by a single probeset, but over 30% of them are targeted by more than one. Some exons are targeted by up to 34 probesets.

While most exons are targeted by a single probeset (68.5%), a substantial proportion are targeted by more than one (Figure 5). Each of the probesets in *list 2 *was mapped to its target exons, and for each exon, all probesets targeting that exon retrieved. All genes from *list 2 *were found in which at least one probeset showed greater than two-fold differential expression. For these, the median fold change for each exon was calculated. A series of gene-wise summaries were then produced (*list 4*), including exon-median fold-change, the inter-exon variance and the inter-exon coefficient of variance, CV = variance/mean.

By sorting on the latter, differentially expressed genes with high consistencies in the measured fold changes for each exon can be found (that is, those that are changing but are unlikely to be alternatively spliced between samples), and those where the CV is high. This latter set has large variations in the fold changes reported for each exon and can be considered to represent putative alternative splicing events. In *list 4 *there are 835 genes with CV higher than 0.5.

The exonmap package allows the structure of individual genes to be plotted, and colored according to probeset intensities or fold changes. Figures 3 and 4 show example genes selected according to their inter-exon CV.

## Identifying regions of novel transcription

Many probesets target regions of putative transcription, with varying degrees of prior supporting evidence. By filtering the DE probeset list (*list 1*) to retain intragenic probesets that do not target known exons, or alternatively, to retain only intergenic probesets, novel regions of transcription can be explored. There were 2,125 probesets identified that showed above median expression in either the MCF7 or MCF10A data (*list 5*). Of these, 1,170 were outside known genes (*list 6*) and 955 inside known genes but outside known exons (*list 7*). This latter set was filtered as described above, with an additional filter to remove probesets with low log fold change (<1). These probesets have been translated into a list of genes suspected to include putative or extended exons (*list 8*).

Detailed methods and an R script that produces all lists described above are provided in Additional data file 2.

## Discussion

The XMAP database and browser, and the exonmap R/BioConductor package, constitute a free and integrated solution for the processing of Affymetrix exon arrays, starting from the initial stages of whole experiment summarization and filtering, through to fine-grained transcriptomic analyses of exon expression and of splicing at the level of individual genes.

Visual representations of the complexity and diversity of genome structure is challenging, and numerous solutions have been presented (for example, [30-33]). Like the UCSC [34] and Ensembl browsers, X:MAP provides access to transcript, exon and gene level information along with a web-based mechanism to visualize it. While both the UCSC and Ensembl browsers provide more types of annotation (including contig and assembly information, and mappings between transcripts and proteins), X:MAP itself focuses on exon array annotation, and instead makes these data available via links to other external resources (such as Ensembl). Affymetrix' Integrated Genome Browser (IGB) provides these mappings via a software application, but provides external links only to the UCSC. In addition, X:MAP draws an explicit distinction between probes and probesets that uniquely target the genome in one place and those that match to more than one location, something that is not done by other browsers, although some of this information is made available (for the previous generation of arrays) in NetAffx [35] and ADAPT [14]. Another distinguishing feature of X:MAP is its novel use of the Google Maps API [17] to provide a fully interactive real-time scrolling interface to the underlying data, rather than providing static pages rendered on the fly. This is particularly advantageous when considering a set of adjacent genes that do not fit into a single screen width.

Underpinning both the R package and browser is a relational database storing genome hits for all the probes represented on the array. The commercial software Genomatix' ChipInspector adopts a similar approach, but uses single probes combined with its own analysis methods, rather than depending on the standard probeset groupings defined by Affymetrix. A number of other commercial analysis tools for exon arrays exist, such as Biotique System's XRAY, JMP^® ^Microarray, Partek^® ^Genomics Suite, and Stratagene's ArrayAssist^®^. Many of them are extensions of previously known software products for standard arrays, and their major analytical aims overlap with those available in exonmap and BioConductor/R, that is, differential expression analysis and splicing analysis. None of the commercial software suites allow integration with the analytical libraries of R and all are proprietary and licensed, while the X:MAP database and exonmap are free and open source (available at [18,36], respectively).

Analysis of exon array data relies on a detailed understanding of the relationship between genes, transcripts and exons and their mappings to array probes/probesets. While genome-structure data may also be processed using Ensembl front-ends and APIs, such as EnsMart [20] or BioMart [21], they do not provide the necessary mappings to exon array probes and probesets. Basic information on cross-hybridization (understood as multiple targeting to the genome) may be obtained from NetAffx [35], but X:MAP handles more variants of probeset characteristics. Thus, exonmap is the only package providing access to fine-grained exon array annotation, and it does so in BioConductor, a popular open source data analysis environment. Exonmap allows probesets to be filtered by the genome feature they are targeting so that, for example, only probesets hitting known exons are retained, or to find expressed probesets that hit the genome between known genes or within known genes, but outside known exons. Exonmap is unique in providing these data and, through them, support for this sort of filtering. It is important because genome annotation is incomplete [37] and changing [7] because there is evidence of widespread transcription outside known genes [8,9] and because feature density on these arrays is very high, resulting in a relatively large number of probes capable of hybridizing in more than one place. Many-to-many relationships can be further refined at the exon level, so that probesets that uniquely hit an exon, but with one or more probes that might hybridize to other parts of the genome, if expressed, can also be distinguished. This level of selection is crucial particularly because the evidence supporting different probesets is variable, and should be reflected in the amount of confidence ascribed to each one. X:MAP makes it possible to focus selectively on high confidence, exon specific data or to widen the net to include less well characterized probesets as appropriate for the analysis in hand.

Exonmap is designed to integrate exon array data with the wide variety of data processing standards implemented in BioConductor. BioConductor (and R) have a number of advantages, which, through exonmap, can be exploited for exon array data. Firstly, because they provide a programming language based environment, it is possible to develop standard analysis workflows that can be applied successively to multiple projects. Script-based analysis is particularly useful when analyzing a number of different datasets over an extended period of time, because analyses can be saved and revisited at a later date. In an environment where multiple collaborative projects are being worked on simultaneously, this is important. The fact that R is a statistical programming language means that it also provides access to a large number of analytical techniques that can be applied to expression data with little or no additional programming. This is particularly useful when considering clinical datasets, where access to, for example, Kaplan Meier or Cox models are required, as well as a variety of clustering and classification tools. Additional methods can also be developed as required. This latter point is important; data analysis approaches are continuing to evolve, and an open source environment, such as BioConductor that allows sharing and development of tools and approaches can greatly facilitate these efforts.

## Conclusion

Processing of exon arrays requires not only efficient handling of genome annotations but also algorithmic flexibility, combined with access to the right set of statistical techniques. The first may be achieved using a properly designed database, the latter with a programming language such as R, which provides access to a comprehensive toolbox of statistical approaches and (through BioConductor) many state of the art bioinformatics algorithms. Visualization techniques and data presentation methods are also required.

By combining a fine-grained genome-level annotation database (X:MAP), a genome browser and an R/BioConductor package, the approach described here provides a unique solution to the problem of exon array analysis. Together, the software tools allow a detailed representation of gene structure to be used alongside the diversity of analytical techniques offered by R and BioConductor in a single, open source, integrated solution.

## Additional data files

The following additional data are available with the online version of this paper. Additional data file 1 is a description of RNA preparation and the hybridization protocol. Additional data file 2 is an R script to generate the lists of probesets and genes described in the paper.

## Supplementary Material

Additional data file 1 Description of RNA preparation and the hybridization protocol. Click here for file

Additional data file 2 R script to generate the lists of probesets and genes described in the paper. Click here for file
